# Risk of Invasive Meningococcal Disease in Preterm Infants

**DOI:** 10.1093/ofid/ofae164

**Published:** 2024-04-15

**Authors:** Anna Calvert, Helen Campbell, Paul T Heath, Christine E Jones, Kirsty Le Doare, Anna Mensah, Shamez Ladhani

**Affiliations:** Centre for Neonatal and Paediatric Infection and Vaccine Institute, St George's, University of London, London, UK; St George's University Hospitals NHS Foundation Trust, London, UK; Immunisation and Countermeasures Division, UK Health Security Agency, London, UK; Immunisation and Countermeasures Division, UK Health Security Agency, London, UK; Centre for Neonatal and Paediatric Infection and Vaccine Institute, St George's, University of London, London, UK; St George's University Hospitals NHS Foundation Trust, London, UK; NIHR Southampton Clinical Research Facility and Biomedical Research Centre, Southampton, UK; University Hospital Southampton NHS Foundation Trust, Southampton, UK; Experimental Sciences, University of Southampton, Southampton, UK; Centre for Neonatal and Paediatric Infection and Vaccine Institute, St George's, University of London, London, UK; Makerere University Johns Hopkins University, Kampala, Uganda; Pathogen Immunology Group, UK Health Security Agency, Salisbury, UK; Immunisation and Countermeasures Division, UK Health Security Agency, London, UK; Centre for Neonatal and Paediatric Infection and Vaccine Institute, St George's, University of London, London, UK; St George's University Hospitals NHS Foundation Trust, London, UK; Immunisation and Countermeasures Division, UK Health Security Agency, London, UK

**Keywords:** epidemiology, Meningococcus, preterm

## Abstract

**Background:**

Invasive meningococcal disease (IMD) is most common in the first year of life. We hypothesized that preterm infants may have a higher risk of IMD and more severe disease than term infants. We compared the incidence, demographics, clinical presentation, and outcomes of IMD in preterm compared with term infants during the first 5 years after implementation of a national meningococcal group B vaccine (4CMenB) for infants in England.

**Methods:**

The UK Health Security Agency conducts enhanced national IMD surveillance with detailed follow-up of all confirmed cases in England. Infants aged <1 year (uncorrected for gestational age) with IMD confirmed between 1 September 2015 and 31 August 2020 were included.

**Results:**

There were 393 infant IMD cases (incidence, 12.4/100 000 live births). Among 363 (92.4%) of the infants with known gestational age, the IMD incidence was higher in preterm (<37 weeks’ gestation) than in term infants (18.3/100 000 vs 10.9/100 000; incidence rate ratio [IRR], 1.68 [95% confidence interval, 1.23–2.29]; *P* = .001). The IMD incidence was highest in those born at <32 weeks’ gestation (32.9/100 000; incidence rate ratio for <32 weeks’ gestation vs term, 3.01 [95% confidence interval, 1.73–5.24]; *P* ≤ .001). There were no differences in demographics, clinical presentation, rate of intensive care admission, or case-fatality rate, but preterm infants were more likely than term infants to have ≥1 reported sequela (14 of 39 [35.9%] vs 51 of 268 [19.0%]; *P* = .02).

**Conclusions:**

Preterm infants had a higher incidence of IMD than term infants and the IMD incidence was highest in infants born at <32 weeks’ gestation. Preterm infants also had a higher risk of IMD sequelae.

## Teaser

IMD incidence was significantly higher in preterm than in term infants and highest in those born at <32 weeks’ gestation. Demographics, clinical presentations, disease severity, and case-fatality rates were similar in both groups, but sequelae more common in preterm infants.

## Graphical Abstract

**Graphical Abstract:**
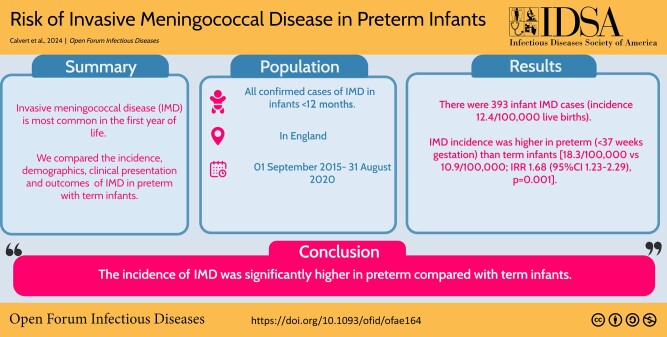
This graphical abstract is also available at Tidbit: https://tidbitapp.io/tidbits/risk-of-invasive-meningococcal-disease-in-preterm-infants

Invasive meningococcal disease (IMD) is associated with significant morbidity and mortality worldwide [1]. There are 13 recognized meningococcal serogroups, of which 6 (A, B, C, W, X, and Y) cause the majority of IMD cases. In the United Kingdom, serogroup B (MenB) is predominant, with most cases occurring in young children, especially in the first year of life [1]. In England, about 7%–8% of infants are born prematurely (at <37 weeks’ gestation) every year. Compared with term infants, preterm infants have a higher risk of infection, severe disease, and worse outcomes from infections [2–6]. For example, they are at greater risk of invasive pneumococcal disease than term infants, likely because of their relative immature immunity and lower maternal transplacental transfer of protective antibodies earlier in gestation [3, 7–9]. Like invasive pneumococcal disease, IMD is dependent on bactericidal antibody for protection, as well as complement factors, many of which are reduced in preterm infants [10]. It is therefore possible that preterm infants, compared with term infants, have an increased risk of IMD and potentially more severe outcomes. The rarity of IMD, however, makes it difficult to identify any significant association with prematurity status.

In September 2015, the United Kingdom introduced a novel recombinant protein-based vaccine against MenB (4CMenB; Bexsero, GSK Biologicals) into the national infant immunization program. The vaccine has been highly effective in preventing IMD due to MenB as well as other meningococcal serogroups in young children [11, 12]. The MenC vaccine was removed from the UK infant schedule in July 2016 following the success of the MenC vaccine program, resulting in very low numbers of MenC cases, and the introduction of MenACWY into the adolescent program.

This study aimed to use enhanced national surveillance to assess IMD risk in preterm compared with term infants and to describe the incidence, risk factors, clinical presentation, and outcomes of IMD in infants aged <1 year during the first 5 years after 4CMenB implementation in England.

## METHODS

### Case Identification

The UK Health Security Agency (UKHSA) conducts enhanced IMD surveillance in England. National Health Service hospital laboratories report all confirmed infections electronically to the UKHSA and submit invasive clinical isolates to the UKHSA Meningococcal Reference Unit for confirmation and strain characterization [13]. General practitioners of patients with confirmed IMD are requested to complete a short questionnaire on demographics, underlying conditions, vaccination status, sequelae, and outcomes. Additional information may be obtained from HPZone, an online platform used by public health practitioners to support case and outbreak management in England [14], or from hospital clinicians, coroners and postmortem reports, as needed. Fatalities are identified through the surveillance questionnaires, the personal demographics service (a national electronic database of National Health Service–registered patients [15]), and electronic death registrations provided by the Office for National Statistics to UKHSA for public health surveillance purposes. Where information about an infant’s gestational age (GA) was not available in the database, strenuous efforts were made to contact the patient's general practitioners to obtain this information.

### Definitions

IMD was defined as detection of *Neisseria meningitidis* from a normally sterile site by means of culture and/or polymerase chain reaction and characterized as 1 of 4 presenting syndromes: meningitis, defined as *N. meningitis* detected in cerebrospinal fluid or *N. meningitidis* detected in blood with clinical features of meningitis; septicemia, defined as *N. meningitidis* detected in blood, without another focus of infection; septic arthritis, defined as *N. meningitidis* detected in joint fluid or in blood with clinical features of septic arthritis; and meningitis with septicemia, defined as the criteria for meningitis being met along with clinical signs of septicemia.

In England, 4CMenB was implemented on 1 September 2015 as a 2-dose schedule at 8 and 16 weeks with a booster at 1 year for infants born on or after 1 July 2015 [16]. As part of a limited catch-up program, infants born in May and June 2015 could receive 4CMenB when they attended their routine 8-week and/or 12-week immunizations [16]. Definitions for vaccine eligibility are summarized in Supplementary Table 1.

### Meningococcal Antigen Typing System

Invasive MenB isolates are routinely subjected to the meningococcal antigen typing system (MATS), which assesses cross-reactivity to 3 vaccine-associated meningococcal surface antigens (factor H binding protein, neisserial adhesin A, and neisserial heparin-binding antigen) alongside genotypic and phenotypic information on porin A to predict 4CMenB coverage [17, 18]. MenB isolates were considered to be covered by 4CMenB if MATS results were positive for any antigens. If <4 antigens were characterized and none were MATS positive, 4CMenB coverage was defined as uncertain. If all 4 antigens were characterized and none were MATS covered, the isolate was defined as not covered.

### Data Analysis

Data were analyzed using Stata software, version 17.0 (StataCorp). The analysis included all IMD cases in infants <1 year of age confirmed between 1 September 2015 and 31 August 2020 in England. Data are mainly descriptive. We described non normally distributed data as medians with interquartile ranges (IQRs) and compared them using the Mann-Whitney *U* test, while proportions were compared using χ^2^ or Fisher exact tests, as appropriate. Population denominators, including number of live births and proportion of preterm births, were obtained from the Office for National Statistics [19]. Incidence rates were calculated as cases per total live births in the 5-year period and are presented as number of cases per 100 000 live births, along with 95% confidence intervals (95% CIs). These were calculated overall and by prematurity status (<37, 32–36^+6^, or <32 weeks’ gestation). Incidence rate ratios (IRRs) with 95% CI were used to compare rates.

To adjust for cases with unknown GA, sensitivity analyses were performed including (1) only cases with known GA; (2) all cases after adjustment for unknown GA, assuming that the same proportion of infants with unknown GA were term as infants with known GA; and (3) all cases but assuming that those with unknown GA were born at term because primary care records are more likely to document preterm births in their records. Performing an analysis with all the infants with missing GAs included in the term birth group will provide the most extreme result for the missing values.

### Ethical Approval

Written consent from the patients was not required. UKHSA has legal permission, provided by regulation 3 of The Health Service (Control of Patient Information) Regulations 2002, to process confidential patient information for national surveillance of communicable diseases, and no additional ethical approval was required for this study.

## RESULTS

During the 5-year surveillance period (1 September 2015 to 31 August 2020), there were 393 IMD cases in infants (incidence, 12.4/100 000); 193 (49.1%) of the infants were female. Ethnicity was reported for 263 infants. The majority (90.1%) were white (n = 237); 3.4% were categorized as “other” (n = 9), 2.7% as mixed race (n = 7), 1.5% as Indian (n = 4), 0.76% as black African (n = 2), 0.76% as Pakistani (n = 2), 0.38% as black Caribbean (n = 1), and 0.38% as Chinese (n = 1). MenB was the most prevalent serogroup (69.7% [n = 274]), followed by MenW (17.6% [n = 69]), MenC (6.1% [n = 24]), MenY (5.1% [n = 20]), and ungrouped strains (1.5% [n = 6]).

### Data on GA

GA was reported for 363 infants (92.4%); 318 of 363 (87.6%) were born at term and 45 of 363 (12.4%) were preterm, including 32 born at 32–36^+6^ and 13 at <32^+0^ weeks’ gestation. After exclusion of those with unknown GA, the IMD incidence was significantly higher in preterm than in term infants (18.3/100 000 vs 10.9/100 000; IRR, 1.68 [95% CI, 1.23–2.29]; *P* = .001). The incidence was higher in those born at <32^+0^ weeks’ gestation (32.9/100 000; IRR vs term infants, 3.01 [95% CI, 1.73–5.24]; *P* ≤ .001) than in those born at 32–36^+6^ weeks’ gestation (15.5/100 000; 1.42 [.99–2.05]; *P* = .06). When infants with unknown gestation were reclassified as term infants according to the proportion of term infants with known GA, IRR remained statistically significant when comparing preterm with term infants overall (1.55 [95% CI, 1.14–2.12]; *P* = .005) and in extremely preterm infants born at <32 weeks’ gestation compared with term infants (2.78 [1.60–4.84]; *P* < .001).

Repeating the analysis assuming that the infants with missing GA were born at term resulted in a slightly higher incidence in term infants (11.95/100 000), with IRR remaining significantly higher in preterm than in term infants (1.53 [95% CI, 1.12–2.10]; *P* = .007) (Table 1). The demographics, clinical characteristics and outcomes of infants with missing GA were similar to those in infants with known GA (Table 2).

**Table 1.** Incidence Rates and Incidence Rate Ratios According to Gestation in Infants Aged <1 Year with Confirmed Invasive Meningococcal Disease in England (September 2015 to August 2020)

GA Category	Incidence Per 100 000 Infants (95% CI) [No. of Cases/No. in Population]	IRR (95% CI) by Definition of Term Population^a^ [*P* Value]
Overall	12.4 (11.2–13.7) [393/3 156 963]	…
Term (≥37 wk GA)	Definition 1, 10.9 (9.7–12.1) [318/2 911 479]; definition 2, 11.82 (10.57–13.06) [344/2 911 479]; definition 3, 11.95 (10.7–13.2) [348/2 911 479]	…
Preterm (<37 wk GA)	18.3 (13.0–23.7) [45/245 484]	Definition 1, 1.68 (1.23–2.29) [.001^b^]; definition 2, 1.55 (1.14–2.12) [.005^b^]; definition 3, 1.53 (1.12–2.10) [.007^b^]
<32 wk GA	32.9 (15.01–50.7) [13/39 551]	Definition 1, 3.01 (1.73–5.24) [<.001^b^]; definition 2, 2.78 (1.60–4.84) [<.001^b^]; definition 3, 2.75 (1.58–4.78) [<.001^b^]
32–36^+6^ wk GA	15.5 (10.2–20.9) [32/205 933]	Definition 1, 1.42 (0.99–2.05) [.06]; definition 2, 1.32 (0.92–1.89) [.14]; definition 3, 1.30 (0.91–1.87) [.15]

Abbreviation: CI, confidence interval; GA, gestational age; IRR, incidence rate ratio.

^a^The following definitions of the term population were used in the analyses: definition 1, the population of infants known to be term; definition 2, the infants known to be term plus the same proportion of infants with missing GA reclassified as term; and definition 3, the infants known to be term plus all infants with missing GA reclassified as term.

^b^Significant at *P* < .05.

**Table 2.** Characteristics of Infants With Invasive Meningococcal Disease According to Gestational Age Category or Missing Status

Characteristic	Infants, % (No. With Characteristic/Total No. With Data for Category)		
	Term (n = 318)	Preterm (n = 45)	Missing Status (n = 30)
Female sex	49.4 (157/318)	46.7 (21/45)	50 (15/30)
White ethnicity	89.8 (194/216)	96.4 (27/28)	84.2 (16/19)
Serogroup			
B	72.0 (229/318)	60 (27/45)	60 (18/30)
C	6.0 (19/318)	8.9 (4/45)	3.3 (1/30)
W	15.1 (48/318)	22.2 (10/45)	36.7 (11/30)
Y	5.7 (18/318)	4.4 (2/45)	0 (0/30)
Ungrouped	1.3 (4/318)	4.4 (2/45)	0 (0/30)
Age at onset category			
1(0–70 days)	22.0 (69/313)	11.1 (5/45)	10 (3/30)
2 (71–126 days)	28.1 (88/313)	37.8 (17/45)	20 (6/30)
3 (127–182 days)	14.4 (45/313)	8.9 (4/45)	23 (7/30)
4 (183–269 days)	18.5 (58/313)	22.2 (10/45)	26.7 (8/30)
5 (270–364 days)	16.9 (53/313)	20 (9/45)	20 (6/30)
Presentation			
Septicemia	50.5 (159/315)	48.9 (22/45)	41.4 (12/29)
Meningitis	33.3 (105/315)	37.8 (17/45)	33.3 (10/29)
Meningitis and septicemia	15.2 (48/315)	13.3 (6/45)	20 (6/29)
Septic arthritis	0.95 (3/315)	0 (0/45)	3.33 (1/29)
PICU status			
Admission	19.1 (54/282)	32.4 (12/37)	13.6 (3/22)
Not admitted	80.9 (228/282)	67.6 (25/37)	86.4 (19/22)
Survival	95.6 (304/318)	97.8 (44/45)	96.7 (29/30)

Abbreviation: PICU, pediatric intensive care unit.

### Clinical Presentation

The median age at onset (IQR) was 132.5 (83–240.5) days for all infants, 126 (78–239) days for term infants, and 154 (88–243) days for preterm infants. Where GA was known, there was no significant difference between term and preterm infants in age at onset (*P* = .24), Most infants presented with septicemia (49.6% [n = 193]), followed by meningitis (33.9% [n = 132]), meningitis with septicemia (15.4% [n = 60]), and septic arthritis (1% [n = 4]). Fever was reported in 256 of 315 infants (81.3%), and 112 of 294 (38.1%) had features of septic shock. The median length of hospital stay (IQR) was 6 (4–9) days. Where GA was known, there were no significant differences between term and preterm infants in any of the following: proportion presenting with fever (*P* = .46), presenting syndrome (*P* = .94), signs of shock (*P* = .57), length of hospital stay (*P* = .48), pediatric intensive care unit (PICU) admission (*P* = .06), or PICU length of stay (*P* = .89) (Table 3).

**Table 3.** Characteristics of Infants With Invasive Meningococcal Disease Overall and by Gestational Age Category When Known with comparison of term and preterm infants

Characteristic	Overall Population		Term		Preterm		*P* Value
	No. With Data	% (No.) With Characteristic^a^	No. With Data	% (No.) With Characteristic^a^	No. With Data	% (No.) With Characteristic^a^	
Age at onset median (IQR), d	388	132.5 (83–240.5)	313	126 (78–239)	45	154 (88–243)	.24^b^
Presentation	389		315		45		
Septicemia only		49.6 (193)		50.5 (159)		48.9 (22)	.94^c^
Meningitis only		33.9 (132)		33.3 (105)		37.8 (17)	
Meningitis and septicemia		15.4 (60)		15.2 (48)		13.3 (6)	
Septic arthritis		1.0 (4)		0.95 (3)		0 (0)	
Fever	315		264		34		
Yes		81.3 (256)		67.3 (214)		60 (27)	.46^d^
No		18.7 (59)		15.7 (50)		15.6 (7)	
Signs of shock	294		246		34		
Yes		38.1 (112)		37.8 (93)		47.1 (16)	.57^d^
No		61.9 (182)		62.2 (153)		52.9 (18)	
Hospital length of stay median (IQR), d	336	6 (4–9)	280	6 (4–9)	36	7 (4–10)	.48^b^
PICU admission	341		282		37		
Yes		20.2 (69)		19.1 (54)		32.4 (12)	.06^d^
No		79.8 (272)		80.9 (228)		67.6 (25)	
PICU length of stay, median (IQR), d	43	4 (2–6)	30	4 (3–5)	10	4.5 (1–9)	.89^b^
Survival	393	95.9 (377)	318	(95.6) 304	45	(97.8) 44	.49^c^
Any sequelae	326	21.2 (69)	268	19.4 (52)	39	35.9 (14)	.04^c,e^
Epilepsy	310	4.8 (15)	257	5.1 (13)	34	5.9 (2)	.63^c^
Other neurological	308	4.5 (14)	257	5.1 (13)	32	3.1 (1)	.30^c^
Amputation	312	1.3 (4)	259	1.2 (3)	34	2.9 (1)	.28^c^
Other	304	7.2 (22)	253	5.9 (15)	33	18.2 (6)	.02^c,e^
Hearing loss	227	14.5 (33/227)	183	12.6 (23)	27	25.9 (7)	.17^c^

Abbreviations: IQR, interquartile range; PICU, pediatric intensive care.

^a^Data represent % (no.) of infants with characteristic unless otherwise specified.

^b^ *P* values calculated with Mann-Whitney *U* test.

^c^ *P* values calculated with Fisher exact test.

^d^ *P* values calculated with χ^2^ test.

^e^Significant at *P* < .05.

### Case-Fatality Rate

There were 16 infant IMD deaths during the surveillance period (case-fatality rate [CFR], 4.1%), with a higher, but statistically nonsignificant, CFR in infants with MenC disease than in other serogroups (MenB, 11 of 274 [4.0%]; MenW, 1 of 69 [1.4%]; MenC, 3 of 24 [12.5%]; MenY, 1 of 20 [5.0%]; and ungrouped, 0 of 6 [0%]; *P* = .20). Where GA was known, CFRs did not differ significantly between preterm and term infants (1 of 45 [2.2%] vs 14 of 318 [4.4%], respectively; *P* = .49).

### Sequelae

Reported sequelae included epilepsy, other neurological sequelae, amputation, hearing loss, and “other”—a category that included skin necrosis/scarring, fatigue, developmental delay, squint, renal pole scarring, visual impairment, and mobility disturbance. At least 1 sequela was reported in 68 of 326 infants (20.9%) who survived their infection and for whom information was recorded, with a higher prevalence in preterm than in term infants (14 of 39 [35.9%] vs 51 of 268 [19.0%]; *P* = .02); preterm infants had significantly more reported sequelae in the “other” category than term infants (preterm, 6 of 33 [18.2%] vs 14 of 253 [5.5%]; *P* = .02) (Supplementary Tables 2 and 3).

### MenC Vaccination Status and Outcomes

None of the 24 infants with MenC disease had received a MenC vaccine; 1 infant was too young to be eligible, and 23 were born after the 12-week infant MenC vaccine dose was withdrawn from the national immunization schedule in July 2016. Of those, 7 of 23 infants would have been too young to be protected from vaccination at 12 weeks of age. Of the 3 infants with MenC who died, all were born after the MenC vaccine dose had been removed from the schedule, 1 of whom was too young to have been eligible for vaccination.

### MenB Vaccination Status, Strain Coverage, and Outcomes

Of the 274 infants with MenB disease, 80 infants were eligible for 1 vaccine dose, 117 were eligible for 2 doses, 74 were ineligible for vaccination, and for 3 infants this information was unknown. Of infants who were eligible for 1 vaccine dose, 62.5% (n = 50) had received this dose, and of those who were eligible for 2 doses 47% (n = 55) had received both doses, 43.6% (n = 51) had received 1, and 9.4% (n = 11) had not received any (Supplementary Table 4). Infants were considered to have received a vaccine dose if disease developed ≥14 days after receipt of the vaccine. There was no evidence that preterm infants were less likely than term infants to be appropriately vaccinated (Supplementary Table 5).

Eleven infants died of MenB disease; 9 of them were unvaccinated (3 were born before 4CMenB implementation, 5 were too young to be vaccinated, and 1 was eligible, but unvaccinated), while 2 were eligible for and had received 1 4CMenB dose. There were no MenB fatalities among 2-dose–vaccinated infants.

Culture results were available for 106 (38.7%) infants with MenB disease (culture only, n = 49; culture/polymerase chain reaction, n = 57), and 46 isolates (43.4%) were MATS covered (1 antigen in 33 isolates, 2 antigens in 9, and 3 antigens in 4), 27 (25.5%) were not covered, and 33 (31.1%) had undetermined coverage (Supplementary Table 6). There was no difference according to MATS status in PICU admission (MATS covered, 10 of 49 [20.4%] admitted to PICU; undetermined coverage, 19 of 84 [22.6%] admitted to PICU; and MATS not covered 7 of 25 [28%] admitted to PICU; *P* = .76) or CFR (MATS covered, 1 of 54 [1.9%] died; MATS undetermined; 2 of 94 [2.1%] died; and MATS not covered, 0 of 27 [0%] died; *P* > .00). Among infants who received ≥1 dose, 40.6% (39 of 96) were infected with MATS-covered isolates (24 of 63 [38.1%] after 1 and 15 of 33 [45.5%] after 2 vaccine doses), compared with 70% of unvaccinated infants (7 of 10) (Supplementary Table 6).

## DISCUSSION

Since the introduction of 4CMenB in England, IMD is rare in infants, but preterm infants have a significantly higher incidence than term infants, with the IMD incidence highest in infants born at <32 weeks. Other characteristics—such as patient demographics, meningococcal serogroup, age at onset, clinical presentation, duration of hospitalization, PICU requirement, and CFR—were similar between preterm and term infants.

MenB remains the most prevalent serogroup causing IMD in this age group, even after 4CMenB implementation. A higher risk of IMD in preterm than in term infants has been reported in 3 case-control studies before 4CMenB licensure [20–22]. In Denmark, the odds ratio for IMD was higher in preterm infants in their first year of life (1.3 [95% CI, 1.1–1.9]) [22]; in France. the odds ratio for hospitalization with IMD was higher in preterm than in term infants (2.7 [1.5–5.0]) [21]; and in the United Kingdom, adolescents had a 3.7-fold higher risk of IMD if they had been born preterm, although this association lost significance when analysis was restricted to microbiologically confirmed cases [20]. While the increase in IMD incidence in preterm infants in the first year of life could be explained by the reduction in complement components and bactericidal antibodies in these infants, the longer-term effects of preterm birth are harder to explain.

Our study is the first to assess IMD risk in infants after 4CMenB implementation, which also provides some cross-protection against other meningococcal serogroups [11, 12]. We also found an increased IMD risk in infants born at the lowest gestations. This is most likely explained by increasing immune immaturity and lower transplacental transfer of protective maternal immunoglobulin G [23, 24].

Some infants in our cohort were too old to be eligible for the MenB vaccine at the start of the program. In addition, infants aged <8 weeks are too young to benefit from vaccination, while those aged 8–16 weeks would have had limited protection from a single dose [12]. Since the introduction of the MenB vaccine, there has been a shift to an earlier age at onset of IMD [25]. This is important, since nearly half of the eligible infants with MenB IMD in our current cohort were infected before they became eligible for their second dose at 16 weeks. Giving the second dose at 12 weeks has the potential to significantly reduce MenB cases further [26]; we are currently investigating the immunogenicity of such an accelerated schedule in a randomized controlled trial in term infants (EudraCT 2021-001561-21).

Timely vaccination is important. We recently reported that more than half the infant MenB IMD cases in England could possibly have been prevented if 4CMenB had been administered on time [26]. Importantly, too, given that the United Kingdom uses a 2-dose infant priming schedule, rather than the licensed 3-dose schedule, it is not known whether preterm infants are as protected with this reduced schedule as term infants; our other randomized controlled trial will help answer this important question (EudraCT 2017-001487-38). Another potential means to prevent a proportion of cases of MenB in young infants would be to introduce an antenatal MenB vaccination program, but the very small number of cases would make such a program very costly, and it is unlikely to be cost-effective. In addition, the lower placental transfer of antibody earlier in pregnancy means that there would be less protection afforded to preterm infants, who are most at risk of IMD.

4CMenB is licensed for prevention of MenB IMD [12], but its protection depends on compatibility between vaccine antigens and surface proteins expressed on the infecting strain, which is serogroup independent. In our cohort, 40.6% of MenB isolates in infants receiving ≥1 4CMenB dose were predicted by MATS to be vaccine preventable, compared with 70% among unvaccinated MenB IMD infants. This is likely a consequence of vaccine protection against MATS-positive MenB strains in vaccinated infants [25]. Among isolates predicted to be vaccine preventable, this coverage was limited to 1 antigen in 71.7% of cases (33 of 46).

There are several possible explanations for breakthrough cases among vaccinated infants. MATS positivity refers to a ≥80% chance of the isolate being killed by immune serum where 1 antigen is present or ≥96% chance of being killed by immune serum where ≥2 antigens are present; it is therefore not designed to express certainty of killing. In addition, since MATS was developed based on a 3 + 1 schedule and not the UK-recommended 2 + 1 schedule, the pooled serum samples used for MATS may overestimate protection [18]. Finally, since ≥2 doses are likely needed for protection, those receiving their first dose only will be substantially less protected [12]. Although a previous study had suggested a higher risk of severe disease in individuals infected with MATS-positive compared with MATS-negative MenB strains [17], this was not seen in our cohort when we used PICU admission and CFR as proxies for severe disease.

We identified 24 children with MenC disease, none of whom had been vaccinated, including 23 born after withdrawal of the 12-week infant MenC dose in July 2016. Of the latter cases, two-thirds may have been prevented if the infant dose had remained. An adolescent MenACWY conjugate vaccine implemented in August 2015, however, has been highly effective in providing both direct and indirect (herd) protection against the 4 vaccine-associated serogroups, such that the numbers of cases due to these 4 meningococcal serogroups remain very low in England [27, 28].

The CFR in our cohort was 4.1%, similar to findings in previous UK studies [29], with no significant difference between term and preterm infants, as was also observed in a previous study on infants with invasive pneumococcal disease [3]. The CFR was highest for MenC disease, but non-MenB IMD case numbers were too small to achieve statistical significance. Reassuringly, rates of severe disease as indicated by PICU admissions, were also similar between preterm and term infants. In contrast, postinfection sequelae in survivors were more prevalent among preterm (35.9%) than term (19.0%) infants. Although hearing loss was the most common complication, which is consistent with other studies on IMD sequelae [30], preterm infants were also more likely to experience sequelae classified as “other,” a range of conditions including skin necrosis and scarring, visual disturbance, and developmental delay. Preterm birth is likely to have played a significant role in the burden of sequelae experienced by the preterm cohort, particularly in terms of developmental delay and visual disturbances [31].

Our study had both strengths and limitations. The comprehensiveness of IMD surveillance in England means that very few cases are likely to have been missed nationally [32]. GA information was obtained for >90% of infants, which strengthens our main assessment of IMD risk in preterm compared with term infants. Furthermore, the increased IRR in the sensitivity analysis in which all infants with missing data were reclassified as term, the most extreme possibility, supports the conclusion that there is a difference in incidence between term and preterm infants. A limitation of our analysis is that the surveillance questionnaire collects limited clinical data on individual cases, so we were unable to perform more detailed analysis on disease severity, laboratory investigations, treatment, or course of illness in our cohort. In addition, our surveillance only extended to 3–6 months after infection and may underestimate long-term complications, especially more subtle neurodevelopmental sequelae. Finally, we could not compare vaccine efficacy in the 2 groups because of a lack of data about vaccine uptake in preterm infants at a population level.

In conclusion, we have identified an increased risk of IMD in preterm infants during the first 5 years after 4CMenB implementation in England. Although disease severity and CFRs were similar, we found some evidence of higher sequelae rates in preterm compared with term infants. Further efforts are needed to reduce the residual IMD burden in infants through timely vaccination, optimization of current vaccine scheduling, and development of next-generation vaccines with broader cross-protection.

## Supplementary Data

Supplementary materials are available at the *Journal of The Pediatric Infectious Diseases Society* online (http://jpids.oxfordjournals.org). Supplementary materials consist of data provided by the author that are published to benefit the reader. The posted materials are not copyedited. The contents of all supplementary data are the sole responsibility of the authors. Questions or messages regarding errors should be addressed to the author.

## Supplementary Material

ofae164_Supplementary_Data

## Notes

***Acknowledgments.*** We are very grateful to Sonia Ribeiro for her work managing the invasive meningococcal disease surveillance database.

***Author contributions.*** A. C., H. C., and S. L. designed the study. A. C. obtained missing data where necessary and analyzed the data (with input from K. L. D. and A. M.). A. C. wrote the initial manuscript draft, and S. L. significantly revised the manuscript for important intellectual content. H. C., P. T. H., C. E. J., K. L. D., and A. M. revised the final manuscript. All authors had final responsibility for the decision to submit for publication.
