# Supplementary material for: Risk of Invasive Meningococcal Disease in Preterm Infants
Source: Open Forum Infect Dis. 2024 Apr 15;11(4):ofae164. doi: 10.1093/ofid/ofae164 (PMC11045171; doi:10.1093/ofid/ofae164)
Supplement: ofae164_Supplementary_Data [file ofae164_supplementary_data.docx]

Supplementary material for OFID

**Supplementary table 1: Summary of the eligibility criteria for 4CMenB and MenC vaccine**

| **Vaccine** | **Status** | **Eligibility criteria** |
| --- | --- | --- |
| 4CMenB* | Ineligible | Born before 01 May 2015 and/or were aged <56 days at disease onset |
|  | For one dose | Date of birth on or after 01 May 2015 and aged ≥56 days (8 weeks) at disease onset |
|  | For two doses | Born on or after 01 June 2015 and aged ≥112 days (16 weeks) at disease onset |
| Men C vaccine** | For one dose | Born before 01 June 2016 and aged ≥84 days (12 weeks) at disease onset |

*Eligible infants were considered appropriately vaccinated with the relevant dose if they developed MenB disease at least 14 days after their last 4CMenB dose.

**Eligible infants were considered appropriately vaccinated if they developed MenC disease at least 14 days after receiving MenC vaccine.

**Supplementary table 2: Sequelae experienced by infants with IMD according to gestational category**

|  | **Number of infants**  **% (n)** | **Term**  **% (n)** | **Preterm**  **% (n)** | **P value^** |
| --- | --- | --- | --- | --- |
| Any sequelae | 20.9 (68/326) | 19.0 (51/268) | 35.9 (14/39) | **0.02** |
| Epilepsy | 4.8 (15/310) | 5.1 (13/257) | 5.9 (2/34) | 0.63 |
| Other neuro | 4.5 (14/308) | 5.1 (13/257) | 3.1 (1/32) | 0.30 |
| Amputation | 1.3 (4/312) | 1.2 (3/259) | 2.9 (1/34) | 0.28 |
| Other ** | 7.2 (22/304) | 5.5 (14/253) | 18.2 (6/33) | **0.02** |
| Hearing loss | 14.5 (33/227) | 12.6 (23/183) | 25.9 (7/27) | 0.17 |

**For details see supplementary table 3; ^Fisher’s exact test

**Supplementary table 3: Details of sequelae reported in category of ‘other’ according to gestational category**

|  | Number of infants | | |
| --- | --- | --- | --- |
|  | **Total**  **(n=21)** | **Term**  **(n=14)** | **Preterm**  **(n=6)** |
| Skin necrosis/scarring | 7 | 5 | 2 |
| Mild fatigue | 1 | 0 | 1 |
| Developmental delay | 6 | 3 | 2 |
| Squint | 1 | 1 | 0 |
| Renal pole scarring | 1 | 1 | 0 |
| Visual impairment | 1 | 1 | 0 |
| Mobility disturbance | 2 | 1 | 1 |
| No details | 2 | 2 | 0 |

**Supplementary table 4: Summary of number of doses of 4CMenB received according to number of doses for which an infant was eligible (all infants with serogroup B disease)**

| N (% of eligible infants who received the indicated number of doses) | | | Received | | | | | |
| --- | --- | --- | --- | --- | --- | --- | --- | --- |
|  |  |  | 0 | | 1 | | 2 | |
|  |  |  | T | PT | T | PT | T | PT |
| Eligible  (n=251) | 0 | T | 59 (100%) |  |  | |  | |
|  |  | PT |  | 6 (100%) |  |  |  |  |
|  | 1 | T | 24 (35.3%) |  | 44 (64.7%) |  |  | |
|  |  | PT |  | 4 (44.4%) |  | 5 (55.6%) |  |  |
|  | 2 | T | 8 (8.4%) |  | 41 (43.2%) |  | 46 (48.4%) |  |
|  |  | PT |  | 0 (0%) |  | 6 (54.5%) |  | 5 (45.5%) |
|  | UK | T | 1 (33.3%) |  | 2 (66.7%) |  | 0 (0%) |  |
|  |  | PT |  |  |  |  |  |  |

| N (% of eligible infants who received the indicated number of doses) | | Received | | |
| --- | --- | --- | --- | --- |
|  |  | 0 | 1 | 2 |
| Eligible  (n=274) | 0 | 74 (100%) |  |  |
|  | 1 | 30 (37.5%) | 50 (62.5%) |  |
|  | 2 | 11 (9.4%) | 51 (43.6%) | 55 (47%) |
|  | UK | 1 | 2 | 0 |

UK= unknown

**Supplementary table 5: Summary of number of doses of 4CMenB received according to number of doses for which an infant was eligible (all infants with serogroup B disease for whom gestational age information was known)**

PT= Preterm; T= Term; UK= Unknown

**Supplementary table 6: MATS coverage of infecting strains according to number of vaccines received**

|  | **Vaccines received** | | |
| --- | --- | --- | --- |
| **MATS coverage** | **0** | **1** | **2** |
| Not covered | 2 | 16 | 9 |
| Unsure | 1 | 23 | 9 |
| Covered | 7 | 24 | 15 |
